# Thrombotic Microangiopathy as a Life-Threatening Complication of Long-Term Interferon Beta Therapy for Multiple Sclerosis: Clinical Phenotype and Response to Treatment—A Literature Review

**DOI:** 10.3390/jcm13061598

**Published:** 2024-03-11

**Authors:** Marco Allinovi, Tommaso Mazzierli, Selene Laudicina, Luisa Pastò, Emilio Portaccio, Maria Pia Amato, Giorgio Trivioli

**Affiliations:** 1 Nephrology, Dialysis and Transplantation Unit, Careggi University Hospital, 50134 Florence, Italy; tommaso.mazzierli@unifi.it (T.M.); selene.laudicina@uslcentro.toscana.it (S.L.); 2 NEUROFARBA Department, University of Florence, 50139 Florence, Italy; luisa.pasto@yahoo.it (L.P.); portaccioe@aou-careggi.toscana.it (E.P.); mariapia.amato@unifi.it (M.P.A.); 3 Department of Nephrology, Cambridge University Hospital NHS Foundation Trust, Cambridge CB2 0QQ, UK; giorgiotrivioli@gmail.com

**Keywords:** interferon beta, thrombotic microangiopathy, malignant hypertension, eculizumab, multiple sclerosis

## Abstract

Thrombotic microangiopathy (TMA) has been observed in some patients receiving interferon beta (IFNβ) therapy for relapsing-remitting multiple sclerosis, but little is known about its clinical features and outcomes. We searched the literature to identify cases with IFNβ-related TMA and assessed their pattern of organ involvement, the presence of prodromal manifestations, the treatments used, and the outcomes. Thirty-five articles met the inclusion criteria, and data of 67 patients were collected. The median duration of IFNβ therapy before the diagnosis of TMA was 8 years, and 56/67 (84%) presented with acute kidney injury (AKI), of which 33 required acute dialysis. All but three patients had manifestations during the four weeks before TMA onset, including flu-like symptoms, headache, and worsening blood pressure control. In only two patients, ADAMTS13 activity was reduced, while 27% had low C3 levels. However, none showed causative genetic mutations associated with development of atypical hemolytic uremic syndrome. All patients discontinued IFNβ, 34 (55%) also received plasma exchange, and 12 (18%) received eculizumab. Complete renal recovery was achieved by 20 patients (30%), while 13 (20%) developed end-stage renal disease. Among those with AKI requiring dialysis, eculizumab therapy was associated with a significantly reduced risk of ESRD compared with plasma exchange. Therefore, TMA with features of aHUS mainly occurs after prolonged treatment with IFNβ and is preceded by prodromes, which may lead to an early diagnosis before life-threatening complications occur. Eculizumab appears beneficial in cases with severe kidney involvement, which supports a role of the complement system in the pathogenesis of these forms.

## 1. Introduction

Multiple sclerosis (MS) is the most frequent inflammatory demyelinating disease of the central nervous system, usually affecting young adults. The treatment of MS has substantially evolved over the last 20 years. Most immunosuppressive treatments have proven beneficial and have been approved mainly for the treatment of relapsing-remitting MS (RRMS) [1], while the management of the progressive forms remains challenging [2]. First-generation injectable disease-modifying therapies in RRMS include interferon beta (IFNβ) 1a or 1b and glatiramer acetate. Despite the well-known efficacy and long-term tolerability of IFNβ, a growing number of reports have recently associated long-term IFNβ therapy with the development of systemic adverse effects that affect multiple organs, including the kidneys, the skin, and the bone marrow [3,4]. The occurrence of such adverse effects is associated with both drug serum levels [5] and the duration of the treatment [6]. The kidney is particularly involved in IFNβ side effects with several patterns of toxicity ranging from isolated hypertension [6] to immune-mediated complications, such as podocytopathies, glomerulonephritis, acute tubular necrosis, (acute and chronic) tubulo-interstitial nephritis, or thrombotic microangiopathy (TMA) [7,8,9]. 

The occurrence of TMA has been described in a few patients under treatment with IFNβ, mostly as individual case reports or small case series [9,10,11]. Intriguingly, a recent study described a dose-dependent toxic effect of the IFNβ on the microvasculature [12], suggesting a role of this therapy in endothelial damage, which may result in TMA, thus challenging the hypothesis of a fortuitous association. Furthermore, a growing number of therapies has been associated with TMA, including calcineurin and mTOR inhibitors [13]. TMA is characterized by thrombocytopenia, microangiopathic hemolytic anemia and signs of ischemic damage in different organs (such as kidney involvement). Notably, it is increasingly recognized that TMA may present with hypertension or renal function impairment with no or mild thrombocytopenia and microangiopathic hemolytic anemia [13,14,15]. Awareness of the full spectrum of TMA is needed in order to recognize it in a timely manner and start treatment before serious complications or death occurs [16].

As the presentation and timing of TMA associated with IFNβ varies widely, it is possible that multiple pathophysiologic mechanisms are involved, such as (i) an increased expression of markers of platelet activation, which has been described in patients with MS [17]; (ii) the shift from the physiological antithrombotic profile of the endothelium to an exaggerated antiangiogenic and antifibrinolytic activity through the inhibition of the VEGF gene transcription, which may be promoted by type 1 IFNs [12]; (iii) activation of the thromboxane receptor and increased production of inflammatory mediators in response to IFNs [18]; (iv) dose-dependent microvascular pathological changes correlated with transcriptional activation of the IFN response through the type I interferon α/β receptor (IFNAR) [19]; (v) direct damage of endothelial cells through the induction of antibodies, such as anticardiolipin or anti-ADAMTS13 antibodies; (vi) the mutual activation of the complement and IFN pathways, resembling an “interferon-complement loop” [20]; (vii) IFNβ-induced podocyte loss and death together with the suppression of podocyte progenitor regeneration [21], leading to proteinuria, hypertension, and glomerulosclerosis [7,22,23], which can subsequently trigger a glomerulopathy-related TMA [24].

The estimated incidence of IFNβ-related TMA in patients with MS was reported to be 7.2 per 100,000 patient-years [25], but this figure is expected to increase as the number of MS patients treated with IFNβ for more than 6 to 10 years has been growing. The real incidence of IFN-related TMA remains undetermined, and it seems plausible that many cases are not recognized. Furthermore, its prognosis remains largely unclear. To improve our understanding of IFNβ-related TMA, we conducted a review of the literature and studied risk factors for its development, its clinical prodromes and presentation at onset, the efficacy of the available treatments, and the disease outcome, with particular reference to kidney failure. 

## 2. Materials and Methods

### 2.1. Literature Review

The PubMed databases were searched in order to identify publications about INFβ-associated TMA in MS from 1999 to 2023, using the following medical subject heading terms: “thrombotic microangiopathy”, “interferon”, “interferon-beta”, “multiple sclerosis”, “haemolytic uremic syndrome”, and “thrombotic thrombocytopenic purpura”. We reviewed all articles written in English, and we selected those that provided individual data of patients with IFNβ-associated TMA. We collected information on drug dose, duration of therapy, manifestations in the week or months before hospital admission, laboratory tests (hemoglobin, platelet, serum creatinine, 24 h proteinuria, and C3 and C4 levels) at onset and throughout follow-up, genetic analysis, treatment performed (plasma exchange, eculizumab or other immunosuppressive drugs), and kidney outcome at last follow-up.

### 2.2. Author Survey

Moreover, we conducted a survey and asked all authors of the selected articles to provide additional information regarding genetic analysis (underlying causative mutations in complement genes), activation of serum complement pathways, renal biopsy features, clinical prodromes during weeks before TMA onset, response to therapy, and long-term outcome.

### 2.3. Definitions

− Thrombotic microangiopathy (TMA) is defined as a disorder characterized by endothelial cell injury and activation with thrombotic occlusion of the small vessels that results in consumptive thrombocytopenia, microangiopathic hemolytic anemia and signs of ischemic damage in different organs [13]. The kidney is frequently involved as a result of endothelial cell injury and thrombosis of the glomerular capillaries. Laboratory findings suggestive of TMA include anemia, increased serum lactate dehydrogenase (LDH), haptoglobin consumption and schistocytes (erythrocyte fragmentation) on a blood film due to hemolysis, decreased platelet count, and kidney injury. If clinical features of TMA are observed, further testing of complement factor levels, such as C3 and C4, as well as CH50 and APH50 is recommended along with tight monitoring of blood pressure and fluid status. Negative results of ADAMTS13 activity and Shiga-toxin research allow one to rule out thrombotic thrombocytopenic purpura and typical hemolytic uremic syndrome, which are two distinct causes of TMA.
− Malignant hypertension (MHT) is defined as a sudden and severe increase in systemic blood pressure (systolic pressure ≥ 180 mmHg and/or diastolic pressure ≥ 120 mmHg) associated with advanced bilateral hypertensive retinopathy and signs or symptoms of acute, ongoing organ damage.
− Posterior reversible encephalopathy syndrome (PRES) is a clinico-radiological syndrome characterized clinically by visual disturbance, headache, seizure, and altered mental status and radiologically by white matter vasogenic edema affecting the posterior occipital and parietal brain regions, bilaterally.

### 2.4. Statistical Analysis

Finally, we performed a descriptive analysis and a multivariable logistic regression analysis including age at onset, malignant hypertension, anticomplement therapy (eculizumab), and plasmapheresis as variables in order to identify independent predictive parameters of renal failure. Categorical variables were reported as absolute frequencies and percentages, while continuous variables were reported as median value and interquartile range (IQR). A *p*-value < 0.05 was considered statistically significant. Statistical analysis was performed using the SPSS 22.0 software package (IBM, Armonk, NY, USA).

## 3. Results

### 3.1. Patients

We retrieved 52 publications and included 35 articles describing a total of 67 cases of IFNβ-related TMA (Table 1). Two were observational studies, and no randomized controlled trials were included. The remaining articles were individual case reports or small case series. All the cases are reported in Table 1.

The main features of patients are presented in Table 2. The median age at diagnosis of INFβ-related TMA was 42 years (IQR 37–52), and the median length of therapy before TMA was 8 years (IQR 5–11.25 years). Forty-one (62%) received INFβ alpha isoform, while 26 (38%) received IFNβ beta isoform. The median dose was 88 μg per week (IQR 73–98). In the entire cohort, all patients had normal kidney function before starting IFNβ with no medical history except for cases with RRMS.

### 3.2. Onset of TMA and Prodromal Manifestations

**Figure 1:**
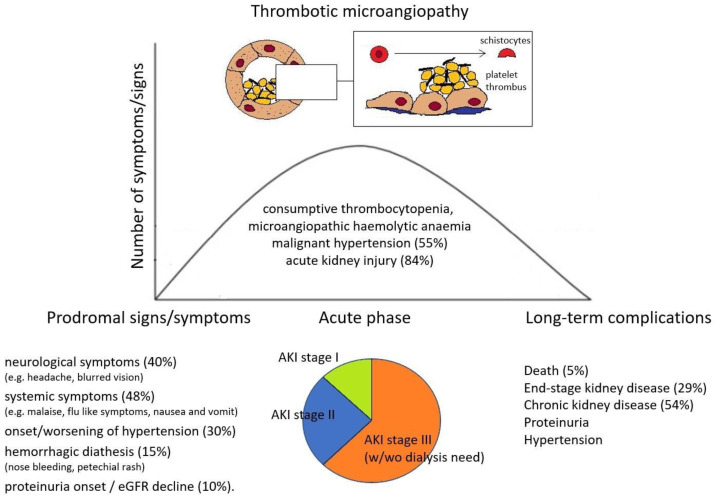
Prodromes, clinical features, and outcomes in IFNβ-related TMA patients.

Forty-eight patients had data regarding manifestations in the previous weeks before the onset of TMA (Table 1). A wide range of symptoms were reported as possible prodromes in the previous month before TMA diagnosis. The main ones were (i) systemic symptoms (malaise, flu-like symptoms) in 23/48 (48%); (ii) neurological symptoms (headache, blurred vision) in 19/48 (40%); (iii) onset or progressive worsening of hypertension in 14/48 (30%); (iv) hemorrhagic diathesis (nose bleeding, petechial rash, etc.) in 7/48 (15%); (v) shortness of breath in 7/48 (15%); (vi) gastrointestinal symptoms (nausea, vomiting, and diarrhea) in 5/48 (10%); and (vii) proteinuria onset and progressive eGFR decline in 5/48 (10%) (Figure 1).

Only three patients (3/48, 6%) presented with a sudden onset of TMA without preceding clinical or laboratory manifestations. In all cases reported in the literature, the onset of IFNβ-related TMA was characterized by systemic involvement, while none had single-organ involvement, such as renal-limited TMA forms (without hemolytic anemia or thrombocytopenia) described in cases induced by VEGF and TK inhibitor [14,15]. Of the 26 patients with available data on anti-ADAMTS13 antibodies, two (8%) had positive anti-ADAMTS13 IgG. Their occurrence was likely due to IFNβ treatment, as suggested by several case reports describing anti-ADAMTS13 IgG after IFN-alpha therapy [33]. The presence of decreased C3 levels at TMA onset was found in 6/22 patients (27%), while predisposing genetic variants in complement regulatory genes were described in 6/25 (24%). In all published cases in which genetic analysis of complement factors was performed, causative mutations were identified in only one case (1/25, 4%) [44].

### 3.3. Organ Involvement

Most patients (56/67, 84%) presented with AKI with a median creatinine at onset of 3 mg/dL (IQR 2.5–4.8 mg/dL). Moreover, 9/67 (13%) had isolated urinary abnormalities without AKI, while two patients had no kidney involvement, only exclusive neurological involvement (2/67, 3%). Most patients with kidney involvement also exhibited an active urinary sediment with hematuria (25/42 patients, 59%) and proteinuria (41/45 patients, 91%). Proteinuria was in the nephrotic range in 31/41 (76%) patients. Among patients with AKI, 6 had stage 1 (11%), 17 had stage 2 (30%), and 33 had stage 3 (59%) according to AKI staging made by KDIGO guidelines (Figure 1). All patients with stage 3 AKI required renal replacement therapy. Furthermore, 34/62 (55%) presented with malignant hypertension (MHT), of whom 8 (23%) had posterior reversible encephalopathy syndrome (PRES). In general, neurological involvement was described in 21/62 (33%) patients. Extra-renal and extra-neurological organ involvement of TMA is reported in one patient with hepatic involvement and in 6/62 (10%) patients with moderate-to-severe heart disease.

### 3.4. Treatment and Outcome

In all patients, IFNβ was withdrawn. In addition to this, those cases with slower responses and more severe manifestations also received plasma exchange or plasmapheresis (34/62, 55%), corticosteroids (29/62, 47%), and/or eculizumab (12/62, 19%). In order to evaluate the efficacy of these adjunctive treatments for INFβ-related TMA and considering the absence of prospective studies, we performed a multivariate analysis on the 33 patients who experienced AKI stage 3 and had available follow-up data. In a multivariable logistic regression analysis including age at onset, malignant hypertension, eculizumab therapy, and plasmapheresis, eculizumab therapy was independently associated with kidney failure at last follow-up as a protective factor (OR 0.14, 95% CI 0.02–0.97, *p* = 0.04), while plasmapheresis did not show any significant association (Table 3).

Considering only those patients who presented with AKI, 16/56 (28%) patients progressed to ESRD. Moreover, 30/56 (53%) patients developed CKD ≥ 3A stage, and 10/56 (18%) had a complete recovery of renal function. Of those without AKI at onset, 6/9 (66%) experienced a complete renal recovery (in terms of proteinuria and hypertension), 2/9 (22%) developed stage 2–3 CKD, and none progressed to ESRD. Overall, 3/67 patients (5%) died. 

## 4. Discussion

Interferon therapy is associated with a wide range of renal manifestations, including isolated proteinuria; podocytopathies, particularly in patients with G1/G2 risk alleles in the APOL1 gene [56]; and TMA [7,8,9]. However, although several mechanisms have been proposed, it remains unclear how IFNβ determines endothelial damage underlying TMA.

Our literature review shows that INFβ-associated TMA most commonly occurs as a life-threatening condition and shares the features of atypical hemolytic uremic syndrome (aHUS), including severe hypertension and renal and neurological involvement [10]. Importantly, almost all cases published in the literature presented with prodromal manifestations likely related to endothelial damage due to interferon, such as hypertension, neurological and constitutional symptoms, and urinary abnormalities (Figure 1). These symptoms should be carefully considered in patients under INF therapy and should raise the suspicion of TMA before more severe complications (such as malignant hypertension, severe AKI, neurologic involvement, and hemolytic anemia) arise. 

Furthermore, patients with INFβ-induced TMA showed a significant risk of progression to ESRD, particularly if they had AKI at onset. Conversely, patients with isolated proteinuria and/or hematuria without AKI reported a high incidence of complete remission after IFNβ discontinuation, significantly higher than that of the AKI group.

Endothelial damage due to IFN has been found to be dose dependent [12], supported by the finding that the median duration of IFN therapy before TMA onset was 8 years in the entire population. According to the classification criteria of drug-induced TMA (DITMA) [57], these INFβ-induced forms appear to be mainly “dose- and/or duration-related toxic reactions” rather than “immune-mediated reactions”. Consistently, chronic renal vascular histopathological abnormalities are frequently reported in numerous case reports of full-blown TMA [22,43]. 

The high prevalence of prodromal signs/symptoms supports the hypothesis that INF-mediated kidney damage is progressive and emphasizes the importance of the awareness of prodromal manifestations (“red flags”) of TMA that could guide clinicians to suspect it before the occurrence of AKI, neurological impairment, and other manifestations of full-blown TMA. In patients on IFNβ with new-onset proteinuria and/or hypertension, the immediate withdrawal of the drug may prevent the development of the severe complications of TMA.

INFβ-associated TMA is characterized by severe kidney involvement, and most patients had stage 3 AKI with variable degrees of proteinuria and/or hematuria. Blood pressure levels were also frequently elevated, and a significant proportion of patients experienced malignant hypertension and PRES, which are likely secondary to the severe endothelial damage in the kidneys. Furthermore, in view of the high frequency of central nervous system involvement, we also collected data on ADAMTS13 activity and anti-ADAMTS13 antibody measurements, which are performed to identify TTP [13,58]). In this cohort, positive anti-ADAMTS13 antibody and reduced ADAMTS13 activity were reported in only two patients. As those patients benefit from an early start of plasma exchange or plasmapheresis, the PLASMIC score should also be calculated while awaiting ADAMTS13 results [59]. 

As recommended for other TMA forms [13,60], complement activation should be evaluated by checking serum C3 and C4 levels. In patients with INFβ-related TMA, no patient presented decreased C4 levels, but 27% had reduced C3 activity. This suggests that the complement alternative pathway is overactivated in a subset of patients, as already shown in other forms of secondary TMA [61]. As suggested by a recent literature review [13], the presence of hypocomplementemia C3 in DITMA is associated with a higher deposition of complement protein in kidney tissue, serves as a major marker of complement activation, and could be used to predict a higher probability of response to complement inhibitor therapy. Among the 25 patients who had genetic testing, only one causative mutation associated with the development of TMA (being so defined as atypical hemolytic uremic syndrome), and only a minority of these showed variants in complement regulatory genes. Hence, similar to other DITMAs [62], in IFNβ-related TMA, the detrimental role of the complement system does not seem to be driven by a causative genetic defect. Rather, predisposing genetic variants in complement regulatory genes in combination with triggering environmental factors, such as IFNβ, may cause complement hyperactivation and consequently complement-mediated TMA (aHUS).

Although the cornerstone of INFβ-related TMA is withdrawal of the causative drug, this is frequently not sufficient to ameliorate kidney function and hematological parameters. Several patients in this cohort were also treated with adjunctive therapies, such as plasmapheresis, immunosuppressive drugs, and/or eculizumab. No prospective clinical trials have been performed to evaluate the effectiveness of those approaches in DITMA. However, among patients with severe IFNβ-related TMA and stage 3 AKI, eculizumab seems to be associated with a lower incidence of ESKD, which further support the role of the complement system in the pathogenesis of these forms. Therefore, eculizumab could represent an effective therapy for INFβ TMA forms, like that noted for other severe secondary TMA forms [11,63]. 

The retrospective nature of all included studies, the small number of reported patients (mostly as individual case reports or small case series), and the scarceness of comparative studies between different potential therapies limit the generalizability of our reported results.

## 5. Conclusions

INFβ-related TMA is a rare but severe adverse complication of prolonged INFβ therapy with a high risk of CKD and ESKD progression. The active surveillance of early signs of kidney involvement through the evaluation of urine, renal function, and blood pressure should be performed in all patients receiving long-term IFNβ therapy at their routine clinic appointments, and abnormalities should lead to promptly suspect TMA. An early withdrawal of the drug may prevent the development of the most serious manifestations of full-blown TMA, thus allowing for a better overall and renal prognosis. This form seems to be complement mediated. In cases of severe full-blown TMA and/or cases of incomplete response to drug withdrawal, eculizumab should be considered as a rescue therapy.

## Figures and Tables

**Table 1:** Reported Cases of INFβ-related TMA in literature.

Article	Gender, Age	Exposure to INFβ	Clinical Prodromes	Clinical Onset	Laboratory Analysis	Complement/ADAMTS13 Activity	Renal Biopsy	Therapy (in Addition to IFNβ Withdrawal and Antihypertensive Drugs)	Renal Outcome
Herrera et al. [26]	F, 44	2 weeks Avonex (IFNβ-1a) 30 mcg/wk;	Fever, petechial rash	Edema, delirium	AKI, proteinuria	----	TMA	PT, steroids	ESRD
	F, 24	4 weeks Avonex (IFNβ-1a) 30 mcg/wk im	Fever, rash, malaise, myalgia	NA	AKI	----	----	PT, steroids	Recovery
Serrano et al. [27]	M, 50	9 years IFNβ-1b 250 mcg/48 h sc	Asthenia, headache, abdominal pain, diarrhea	Headache, lipothymia, visual loss	AKI	NA	NA	PT, steroids	Recovery
Li Cavoli et al. [28]	F, 36	3 months IFNβ-1a 22 μg tw sc	Asthenia, myalgia, dyspnea, weight gain	Hypertension pleuro-pericarditis	AKI, microhematuria nephrotic syndrome	No complement mutations	Chronic TMA IF: mesangial IgM, C1q, and fibrinogen	PT, steroids	ESRD
Broughton et al. [29]	F, 53	8 years Betaseron (IFNβ-1b) 250 mcg/48 h sc	Mild hypertension	Hypertension, headache	AKI, proteinuria hematuria	Normal C3 and C4 levels	TMA IF: C3++	----	CKD
Bensa et al. [30]	M, 45	5 years IFNβ-1a	NA	NA	Nephrotic proteinuria	----	NA	----	Recovery (mild proteinuria)
Olea et al. [31]	F, 37	5 months IFN-β	Fatigue, arthralgia	Hypertension	AKI, proteinuria	No complement mutations	Chronic TMA IF: fibrinogen +	Steroids	Recovery
Tavakoli et al. [32]	M, 30	3 months Rebif (IFNβ-1a) 44 μg tw sc	Massive epistaxis, ecchymosis	Normal BP	AKI	Normal C3 and C4 levels	Normal	Steroids	Recovery
Modrega et al. [22]	F, 28	10 years Betaseron (IFNβ-1b) 250 mcg/48 h sc	Hypertension, headache	Hypertension	Nephrotic proteinuria	---	Chronic TMA	----	Recovery
Nerrant et al. [33]	F, 38	5 years IFNβ-1a 22 μg tw sc	Asthenia, lower limb edema	Hypertension, PRES	AKI	Normal ADAMTS13 Normal C3 and C4 levels, no complement mutations	Acute TMA IF: IgM	PT, steroids	ESRD
Mahe et al. [34]	F, 38	5 years Rebif (IFNβ-1a)	Asthenia, cramps, headaches, anorexia, anemia	Hypertension, headache	AKI	Normal C3 and C4 levels, no complement mutations	Acute and chronic TMA	----	CKD
Mahe et al. (FPD)	NA, 58	5 years	NA	Hypertension	AKI	----	TMA	PT, steroids	CKD
	NA, 66	1 year	NA	Hypertension	AKI	----	TMA	----	Remission
	NA, 52	>3 years	NA	Hypertension	AKI	ADAMTS13 deficit	TMA	PT	CKD
	NA, 55	6 years	NA	NA	NA	Anti-ADAMTS13 Ab	TMA	PT, steroids, rituximab	Remission
	NA, 38	10 years	NA	NA	NA	-----	TMA	PT, steroids	NA
Orvain et al. [35]	M, 52	4 years IFN-β	Headaches, hypertension, Raynaud’s phenomenon, vision loss	Malignant hypertension, tachycardia, dyspnea	AKI, hematuria, proteinuria, ANA 1:1280	No complement mutations, normal C3 and C4 levels, anti-ADAMTS13 Ab, ADAMTS13 activity 5%	Severe TMA	PT, steroids, rituximab	ESRD
Vosoughi et al. [36]	M, 52	14 years Betaseron (IFNβ-1b) 250 mcg/48 h sc	Shortness of breath, wheezing	Malignant hypertension, generalized tonic– clonic seizures	AKI	---	---	PT	ESRD
	F, 41	11 years Rebif (IFNβ-1a) 44 mcg tw sc	Headache	Malignant hypertension, headache, seizures, PRES	Proteinuria, microhematuria, hyaline cast	---	---	---	Recovery
Rubin et al. [37]	F, 41	10 years IFN-β	NA	Malignant hypertension, PRES seizures	AKI, hematuria, proteinuria	---	---	---	Recovery
Larochelle et al. [38]	F, 34	1,2 years sc IFNβ-1a 44 mcg tw	Asthenia, frontal headache	Malignant hypertension, headache, confusion, left hemiparesis, hemineglect	No AKI, ARDS, vasogenic edema	---	---	PT, steroids, vincristine, rituximab	Death
	F, 47	11 years sc IFNβ-1a 44 mcg tw	---	Malignant hypertension, headache	AKI, proteinuria	---	chronic TMA	PT, steroids	CKD
	F, 41	5 years IFNβ-1a 22 μcg tw sc and then 44 mcg tw sc	Headache, cough, GI discomfort, confusion, upper limb weakness	Hypertension, headache, confusion, seizures, PRES	AKI	---	TMA	PT, steroids, rituximab	ESRD
Capobianco et al. [39]	F, 37	5 years IFN-beta-1a 30 mcg/w im; 8 years 44 mcg tw sc	Arthralgia, peripheral oedema, hypertension	Hypertension, interstitial (sarcoid-like) lung disease	Proteinuria, hematuria	---	Chronic TMA	----	Remission (mild proteinuria)
Hunt et al. [10]	NA	8 years Rebif (IFNβ-1a)	Headache, vomiting, hypertension	Malignant hypertension, headache, seizures	AKI	Normal ADAMTS13 activity, no complement mutations	Chronic TMA	NA	ESRD
	NA	6 years Rebif (IFNβ-1a)	Headache, vomiting	Malignant hypertension, headache	AKI	No complement mutations, normal ADAMTS13 activity	Chronic TMA	NA	ESRD
	NA	10 years Rebif (IFNβ-1a)	---	Malignant hypertension, seizures, confusion	AKI	Normal ADAMTS13 activity, no complement mutations	Chronic TMA	NA	Recovery
	NA	6 years Rebif (IFNβ-1a)	Vomiting	Hypertension	AKI	Normal ADAMTS13 activity no complement mutations	Chronic TMA	NA	ESRD
	NA	NA Rebif (IFNβ-1a)	Nausea, malaise, worsening mobility, hypertension	Hypertension	AKI	NA	TMA	NA	Death
Azkune Calle et al. [40]	M, 36	9 years Rebif (IFNβ-1a) 44 mcg tw sc	Shortness of breath	Hypertension, heart failure with reduced systolic function, pulmonary hypertension, psychosis	Normal renal parameters	---	NA	PT, steroids	Recovery (but resistant hypertension)
Nishio et al. [41]	M, 41	10 years Betaferon (IFNβ-1b) 9,600,000 UI/24 h sc	Hypertension, facial edema, syncopal attack	Hypertension, visual disorder, nausea, truncal ataxia, dysarthria, lethargy, PRES	AKI, proteinuria microhematuria	Normal C3 and C4 levels, ADAMTS13 activity 32%, no ADAMTS13 inhibitors	---	PT, steroids	Recovery
Gerischer et al. [42]	F, 53	9 years IFNβ-1a 30 mg/wk 44 mcg tw sc for 5 yr	Injection site reactions, flu-like symptoms (muscle ache, headaches, shivers, fatigue)	Hypertension, weakness, headache, seizure, PRES	Proteinuria	Low C3 levels and normal C4 levels	---	steroids, PT, rituximab	Recovery
Piccoli et al. [43]	M, 31	11 years IFN-beta-1a	---	---	Proteinuria	Normal C3 and C4 levels	Acute TMA, collapsing glomerulopathy IF: negative	---	Recovery
Milan Manani et al. [44]	F, 48	7 years IFNβ-1b	progressive eGFR reduction, microhematuria, proteinuria, hypertension	severe hypertension, asthenia	AKI, subnephrotic proteinuria, microhaematuria	low C3 level, CFH-H3 haplotype, heterozygous deletion of CFHR1-CFHR3, normal ADAMTS13	---	PT, steroids, eculizumab	CKD
Allinovi et al. [45]	F, 46	15 years Rebif (IFNβ-1a)	Asthenia, bruise	Malignant hypertension, headache, diastolic heart dysfunction	AKI, proteinuria	No complement mutations, normal ADAMTS13	---	PT, steroids, eculizumab	CKD
	F, 32	11 years Betaferon (IFNβ-1b)	Dyspnea	Malignant hypertension, left ventricular hypertrophy	AKI, nephrotic proteinuria	No complement mutations, heterozygous CFHR1-R3 deletion, normal ADAMTS13	---	PT, eculizumab	CKD
	M, 35	14 years Rebif (IFNβ-1a)	Blurred vision, palpitations, gingival hemorrhage, tinnitus	Malignant hypertension, visual loss, tinnitus, headache	AKI, proteinuria, microhematuria	Low C3 levels, heterozygous MCP mutation, normal ADAMTS13	Acute TMA IF: C3+	PT, eculizumab	CKD
Omoto et al. [46]	F, 42	8 years IFN-β1b 250 mcg/48 h sc	Headache, nausea, hypertension	Hypertension, headache, PRES, gastrointestinal involvement	AKI, proteinuria	normal ADAMTS13	---	----	Recovery
Pérez et al. [47]	F, 48	9 years IFN-β1a 44 mcg tw sc	Upper respiratory tract infection	Malignant hypertension	AKI, proteinuria, microhematuria	normal ADAMTS13	Acute TMA	----	CKD
Etemadifar et al. [48]	F, 25	1,8 years IFN-β1a	Fever, nausea	Coma	AKI, microhematuria, acute hepatitis	---	---	PT, steroids	Recovery
Baghbanian et al. [49]	F, 38	10 years IFN-β1a sc	Epistaxis, gingival hemorrhage	Hypertension	AKI, proteinuria	NA	NA	PT, steroids	Recovery
Yam et al. [50]	F, 57	20 years IFN-β1a	Shortness of breath	Malignant hypertension, pulmonary edema	AKI, nephrotic proteinuria	Normal C3 and ADAMTS13 activity	Chronic TMA	----	CKD
Gianassi et al. [8]	F, 55	17 years IFN-β1a (Rebif)	Headache, hypertension	Malignant hypertension	AKI, proteinuria, microhematuria, leukocyturia	Low C3 levels, normal ADAMTS13, no complement mutations	Acute TMA IF: C3++	Eculizumab	ESRD
Malekzadeh et al. [51]	M, 43	9 years im Avonex (IFN-β1a) 30 mcg/wk; 8 years Recigen (IFN-β1a) 44 mcg tw sc	Headache, vomiting, blurred vision	Malignant hypertension	AKI	---	---	PT, steroids, rituximab	ESRD
Parisi et al. [52]	M, 39	12 years im Avonex (IFN-β1a) 30 mcg/wk; 7 years sc Rebif (IFN-β1a) 22 mcg tw then 44	Asthenia, fever	Hypertension, blurred vision, confusion, speech disorder, PRES	AKI	Normal C3 and C4 levels, normal ADAMTS13 activity	Acute TMA IF: IgG +	PT, eculizumab	CKD
Daurvegne et al. [9]	F, 53	NA, Betaferon (IFNβ-1b)	NA	Hypertension	AKI, proteinuria	NA	TMA	steroids	Death
	F, 58	8 years Betaferon (IFNβ-1b)	NA	Hypertension	AKI, proteinuria	NA	TMA	---	CKD
	F, 39	6 years IFN-β1a (Rebif)	Diarrhea, muscle weakness, fatigue rhinopharingitis	Malignant hypertension	AKI, proteinuria, microhematuria	Normal C3, C4, CH50, CFH, CFI, and MCP levels; ADAMTS13 activity 36%; negative anti-CFH ab; CFHR1 homozygous deletion	TMA	PT	CKD
	M, 65	7 years IFN-β1a (Rebif)	NA	Hypertension	AKI	NA	TMA	---	CKD
	F, 37	12 years IFN-β1a (Rebif)	NA	Hypertension	AKI, proteinuria	NA	TMA	PT, steroids	CKD
	M, 52	4 years IFN-β1a (Rebif)	NA	Hypertension	Proteinuria	NA	TMA	---	CKD
	M, 47	4 years IFN-β1a (Rebif)	NA	Hypertension	AKI, proteinuria	NA	TMA + FSGS	eculizumab	CKD
	M, 61	12 years Betaferon (IFNβ-1b)	NA	Hypertension	AKI, proteinuria	NA	TMA	---	CKD
	F, 37	7 years IFN-β1a (Rebif)	NA	Hypertension	Proteinuria	NA	TMA + FSGS	---	Recovery
	F, 48	2 years IFN-β1a (Rebif)	NA	Hypertension	AKI, proteinuria	NA	TMA	PT, steroids	CKD
	F, 38	2 years IFN-β1a (Rebif)	NA	Hypertension	Proteinuria	NA	TMA + FSGS	---	CKD
	M, 42	5 years IFN-β1a (Rebif)	Diarrhea, abdominal pain, vomiting, dyspnea	Malignant hypertension, pulmonary edema, rhabdomyolysis	AKI, proteinuria, microhematuria	Normal C3, C4, CH50, CFH, CFI, and MCP levels; ADAMTS13 activity 26%; negative anti-CFH ab	TMA + FSGS	PT, steroids, eculizumab	CKD, persistent proteinuria
	F, 28	6 years IFN-β1a (Rebif)	Diarrhea, vomiting, headaches	Malignant hypertension	AKI, proteinuria	Normal C3, C4, CH50, CFH, CFI, and MCP levels; ADAMTS13 activity 30%; negative anti-CFH ab	TMA	---	CKD, persistent proteinuria
	F, 52	4 years IFN-β1a (Rebif)	Blurred vision, headaches	Hypertension, pulmonary edema, myocardial ischemia, neurological involvement	AKI, proteinuria	Normal C3, C4, CH50, CFH, CFI, and MCP levels; ADAMTS13 activity 19%; negative anti-CFH ab, CFHR5 variant	TMA	---	CKD
	F, 56	5 years IFN-β1a (Rebif)	Fatigue, weight loss	Malignant hypertension, pulmonary edema myocardial ischemia	AKI, proteinuria microhematuria	Normal C3, C4, CH50, CFH, CFI, and MCP levels; ADAMTS13 activity 25%; negative anti-CFH ab	TMA	steroids	CKD
	F, 56	14 years Betaferon (IFNβ-1b)	NA	Hypertension	AKI, proteinuria	NA	TMA + FSGS	---	CKD
Allinovi et al. [11]	F, 40	15 years IFN-β1a (Rebif)	Fatigue, injection site reactions	Hypertension	AKI	Low C3 levels, normal ADAMTS13, CFH-H3 homozygous haplotype	---	PT, steroids, eculizumab	CKD
	M, 34	13 years IFN-β1a (Rebif)	Headache, flu-like symptoms	NA	AKI	No complement mutations	NA	PT, eculizumab	ESRD
	M, 38	19 years IFN-β1a (Rebif)	NA	Hypertension, fever, hemoptysis	AKI	low C3 level	TMA IF: C3++	PT, eculizumab	CKD
Taghavi et al. [53]	M, 48	13 years IFN β-1a (Avonex) 30 μg weekly, IFN β-1a (Rebif^®^) 44 μg, thrice weekly	No prodromes	Focal epileptic seizure	AKI	Normal C3, C4, CH50, and AP50 levels; elevated factor B, factor Bb, and sC5b-9; normal ADAMTS13; no complement mutations	TMA IF: negative	---	CKD
Mrabet et al. [54]	M, 28	3 years	NA	Malignant hypertension, seizures	AKI	Acquired CFI deficiency	NA	PT, steroids	ESRD
Satori et al. [55]	F, 54	15 years IFN β-1b	Hypertension, blurred vision	Malignant hypertension, retinal bleeding, stroke	AKI, proteinuria	Normal C3, C4, and CH50 levels; ADAMTS-13 activity 28%	NA	---	CKD

Legend: AKI, acute kidney injury; CKD, chronic kidney disease; ESKD, end-stage kidney disease; FSGS, focal segmental glomerulosclerosis; IF, immunofluorescence staining; IFN, interferon; IQR, interquartile; NA, not available; PRES, posterior reversible encephalopathy syndrome; PT, plasma therapy; TMA, thrombotic microangiopathy.

**Table 2:** Characteristics of the study population.

**Clinical Features at TMA Diagnosis:**	
Age (years), median (IQR)	42 (37–52)
Length of IFNβ therapy (years), median (IQR)	8 (5–11.25)
Serum creatinine (mg/dL), median (IQR)	3 (2.5–4.8)
Acute kidney injury, *n* (%)	56/67 (84)
Malignant hypertension, *n* (%)	34/62 (55)
PRES, *n* (%)	8/67 (12)
**Potential etiopathogenetic mechanisms underlying IFN-beta toxicity:**	
IFNβ dose (μg per week), median (IQR)	88 (73–98)
Hypocomplementemia C3, *n* (%)	6/22 (27)
Predisposing variants in complement regulatory genes, *n *(%)	6/25 (24)
Causative mutations in complement regulatory genes, *n* (%)	1/25 (4)
Anti-ADAMTS13 antibodies, *n* (%)	2/26 (8)
**Treatment of TMA** **(in addition to withdrawal of IFN-beta and antihypertensive drugs):**	
Plasma therapy, *n* (%)	34/62 (55)
Corticosteroids, *n* (%)	29/62 (47)
Eculizumab, *n* (%)	12/62 (19)
**Outcome:**	
Renal recovery, *n* (%)	20/67 (30)
CKD, *n* (%)	31/67 (46)
ESKD, *n* (%)	13/67 (19)
Death, *n* (%)	3/67 (4)

Legend: CKD, chronic kidney disease; ESKD, end-stage kidney disease; IFN, interferon; IQR, interquartile; PRES, posterior reversible encephalopathy syndrome; TMA, thrombotic microangiopathy.

**Table 3:** Multivariable regression analysis of parameters associated with renal failure in patients with INFβ-related TMA and AKI stage 3 at onset included in the study cohort.

Features	*p*-Value	OR (95% CI)
Age at onset	0.13	0.91 (0.81–1.03)
Malignant hypertension	0.29	3.38 (0.36–32.2)
Plasmapheresis	0.59	1.77 (0.22–14.12)
Eculizumab	0.04	0.14 (0.02–0.97)

## Data Availability

The data underlying this article will be shared on reasonable request made to the corresponding author.
